# Tissue-specific regulation of epidermal contraction during *Caenorhabditis elegans* embryonic morphogenesis

**DOI:** 10.1093/g3journal/jkab164

**Published:** 2021-05-11

**Authors:** Elizabeth D Drewnik, Tobias Wiesenfahrt, Ryan B Smit, Ye-Jean Park, Linda M Pallotto, Paul E Mains

**Affiliations:** Department of Biochemistry and Molecular Biology, Alberta Children’s Hospital Research Institute, University of Calgary, Calgary, AB T2N 4N1, Canada

**Keywords:** *C. elegans*, morphogenesis, actin, embryo, epidermis, Rho kinase, myosin phosphatase, p21 activated kinase

## Abstract

Actin and myosin mediate the epidermal cell contractions that elongate the *Caenorhabditis elegans* embryo from an ovoid to a tubular-shaped worm. Contraction occurs mainly in the lateral epidermal cells, while the dorsoventral epidermis plays a more passive role. Two parallel pathways trigger actinomyosin contraction, one mediated by LET-502/Rho kinase and the other by PAK-1/p21 activated kinase. A number of genes mediating morphogenesis have been shown to be sufficient when expressed either laterally or dorsoventrally. Additional genes show either lateral or dorsoventral phenotypes. This led us to a model where contractile genes have discrete functions in one or the other cell type. We tested this by examining several genes for either lateral or dorsoventral sufficiency. LET-502 expression in the lateral cells was sufficient to drive elongation. MEL-11/Myosin phosphatase, which antagonizes contraction, and PAK-1 were expected to function dorsoventrally, but we could not detect tissue-specific sufficiency. Double mutants of lethal alleles predicted to decrease lateral contraction with those thought to increase dorsoventral force were previously shown to be viable. We hypothesized that these mutant combinations shifted the contractile force from the lateral to the dorsoventral cells and so the embryos would elongate with less lateral cell contraction. This was tested by examining 10 single and double mutant strains. In most cases, elongation proceeded without a noticeable alteration in lateral contraction. We suggest that many embryonic elongation genes likely act in both lateral and dorsoventral cells, even though they may have their primary focus in one or the other cell type.

## Introduction

Cell-shape changes are key features of biological events ranging from development to disease (Cooper 2000; Munjal and Lecuit 2014). Embryonic development relies on morphogenesis to determine organ and overall body shape. Groups of cells exhibit cytoskeletal rearrangements, often carried out by actin-myosin contractions, resulting in cell-shape changes that drive morphogenesis. A convenient model for studying morphogenesis is the *Caenorhabditis elegans* embryo, which transforms from an ovoid embryo into a long, thin vermiform larva by actin-myosin contractions within the epidermal cells (the nematode hypodermis) (Priess and Hirsh 1986; Chisholm and Hardin 2005; Vuong-Brender *et al.* 2016; Carvalho and Broday 2020).

**Figure 1:**
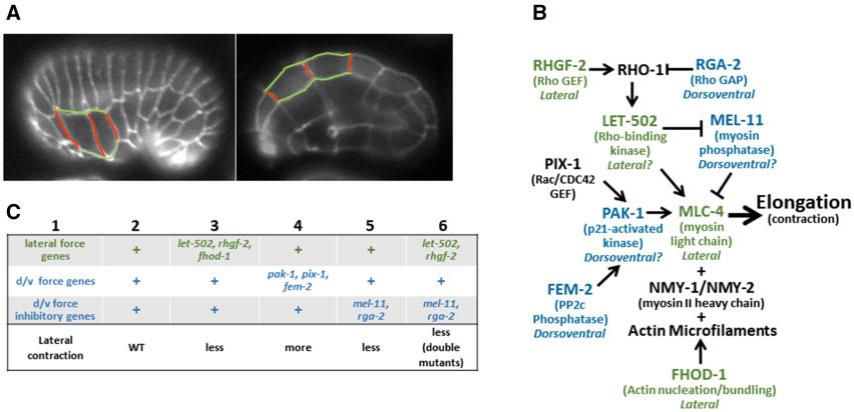
Summary of early elongation in the *C. elegans* embryo. (A) Embryos at the 1.1-fold (left) and twofold (right) stages are shown with selected lateral membranes outlined. Anterior is to the left and dorsal to the top. Contraction of the lateral cells in the dorsoventral axis results in the shortening of the membranes highlighted in red with a lengthening of those marked in green. These cell shape changes drive embryo elongation. (B) Elongation pathway. Those genes for which lateral (green) or dorsoventral (blue) expression has been demonstrated to be sufficient are as indicated. Those that we tested have question marks. (C) Model of elongation proposing that genes are primarily active in either lateral (green) or dorsoventral (blue) epidermal cells. *let-502, rhgf-2*, and *fhod-1* (column 3) are proposed to provide lateral force and so mutants could have less lateral cell shortening at any given stage of elongation. Weaker dorsoventral force in *pak-1, pix-1*, or *fem-2* mutants (column 4) lead to relatively more lateral cell contraction. Stronger dorsoventral force resulting from loss of *mel-11* or *rga-2* (column 5) would result in less lateral shortening. Pairwise double mutants of *let-502* or *rhgf-2* with *mel-11* or *rga-2* (column 6) may have contractile dorsoventral cells and passive lateral cells. These may elongate with less lateral contraction at any given stage of embryo lengthening. A more detailed explanation is included in Supplementary Figure S1.

Prior to the start of elongation, *C. elegans* epidermal cells arise on the dorsal side of the embryo and migrate toward the ventral midline (Williams-Masson *et al.* 1997). After encasing the embryo, the epidermis is composed of two rows of dorsal cells straddling the midline, with a row of lateral (seam) and ventral cells on each side of the embryo. The dorsal cells intercalate to form one single row across the midline, slightly lengthening the top side of embryo to result in the “bean” stage (Sulston *et al.* 1983; Williams-Masson *et al.* 1997). Elongation past this point occurs in two steps. Early elongation from the bean to the twofold stage is driven by nonmuscle actin-myosin contractions of epidermal cells (Figure 1A, “fold” refers to the length of the embryo curled up in the eggshell relative to the length of the long axis of the eggshell). Late elongation continues from the two to the fourfold stage and depends on body wall muscle contraction (Williams and Waterston 1994; Chisholm and Hardin 2005; Vuong-Brender *et al.* 2017; Lardennois *et al.* 2019).

We focus on early elongation, up to the twofold stage. The core players include LET-502/Rho kinase and MEL-11/myosin phosphatase (Figure 1B) (Wissmann *et al.* 1997, 1999; Shelton *et al.* 1999; Piekny *et al.* 2003; Diogon *et al.* 2007; Gally *et al.* 2009). Rho kinase (also referred to as Rho-binding kinase, ROCK, or ROK) is activated by the small GTPase Rho (Sit and Manser 2011; Julian and Olson 2014; Truebestein *et al.* 2016). Among Rho kinase substrates are myosin light chain (MLC-4 in worms), whose phosphorylation leads to the activation of nonmuscle myosin. *mel-11* encodes the regulatory MYPT subunit of myosin phosphatase, and opposes LET-502/Rho kinase activity by the dephosphorylation of myosin light chain. In addition, MEL-11 is inhibited by LET-502, adding a second layer of regulation to activate contraction. Consequently, *let-502*/Rho kinase mutants exhibit hypocontraction and arrest during early elongation, while *mel-11*/myosin phosphatase mutants undergo unregulated hypercontraction and often burst (Wissmann *et al.* 1997, 1999; Diogon *et al.* 2007; Gally *et al.* 2009).

*let-502; mel-11* double mutants grow to adulthood, albeit with a mild dumpy and lumpy phenotype and sterility (Wissmann *et al.* 1997, 1999). Lethality in single mutants results when the system is out of balance. However, the viability of the double indicates that there is a parallel pathway that can mediate elongation when both *let-502* and *mel-11* are absent. Indeed, PAK-1/p21-activated kinase and its activators, FEM-2/PP2c protein phosphatase and PIX-1/Rac GEF regulate contraction in parallel to the LET-502/MEL-11 pathway (Figure 1B) (Piekny *et al.* 2000; Gally *et al.* 2009; Vanneste *et al.* 2013; Martin *et al.* 2014, 2016). LET-502/MEL-11 is likely the primary pathway as their individual null mutants show fully penetrant lethal elongation phenotypes. In contrast, *pak-1, fem-2*, and *pix-1* are viable with each showing weak elongation phenotypes, unless they are in combination with *let-502* to inactivate both pathways (Piekny *et al.* 2000; Gally *et al.* 2009; Vanneste *et al.* 2013; Martin *et al.* 2014, 2016).

Contraction driving embryonic elongation is primarily localized to the lateral subset of epidermal seam cells (Figure 1A). Dorsoventral cells are more passive but generate tension and so remain rigid as the lateral cells pull on them, transmitting the force around the embryo (Priess and Hirsh 1986; Chisholm and Hardin 2005; Martin *et al.* 2016; Vuong-Brender *et al.* 2017; Gillard *et al.* 2019). The contractile apparatus in lateral *vs* dorsoventral cells differs in several respects, contributing to their different behaviors (Figure 1B). Lateral epidermal cells exhibit a meshwork of actin, which is typical of animal cells that undergo shape change (Roh-Johnson *et al.* 2012; Munjal *et al.* 2015) while dorsoventral cells show circumferential actin bundles that stiffen the cells (Priess and Hirsh 1986; Gally *et al.* 2009; Vuong-Brender *et al.* 2017). FHOD-1, a member of the formin class of actin nucleators (Mi-Mi *et al.* 2012), rescues mutants when expressed laterally and so may contribute to the lateral *vs* dorsoventral differences in actin deployment (Vanneste *et al.* 2013; Refai *et al.* 2018). Nonuniform RHO-1 activity across the epidermal cells also appears to contribute to differences between lateral and dorsoventral contraction. The Rho activator RHGF-2/Rho guanine exchange factor (GEF) and the Rho inhibitor RGA-2/GTPase activating protein (GAP) are expressed throughout the epidermis; however, lateral RHGF-2 expression is sufficient for *rhgf-2* viability and dorsoventral RGA-2 expression can rescue *rga-2* mutants (Diogon *et al.* 2007; Lin *et al.* 2012; Chan *et al.* 2015). Several other genes appear to have their focus in either one or the other tissue. Mutants of LET-502*/*Rho kinase show phenotypes in the lateral cells (Martin *et al.* 2016) while lateral activity of its substrate MLC-4 is sufficient for elongation (Shelton *et al.* 1999; Gally *et al.* 2009). In addition, *let-502* transcriptional reporters show higher lateral than dorsoventral expression (Wissmann *et al.* 1999). Mutant *mel-11* dorsoventral cells show excess pulling forces, indicating the wild-type gene is active in those cells (Diogon *et al.* 2007) and *mel-11* transcriptional reporters are higher dorsoventrally. *pak-1* and its activator *pix-1* have phenotypes primarily in the anterior dorsal cells (Martin *et al.* 2014, 2016). Another PAK-1 activator, FEM-2, is sufficient in dorsoventral epidermal cells (Refai *et al.* 2018).

An important observation is that all four pair wise double mutants that lose an activator of contraction that is thought to act in lateral cells (*let-502* and *rhgf-2*) and an inhibitor that is expected to function dorsoventrally (*mel-11* and *rga-2*) are viable even though the individual mutations are lethal (Wissmann *et al.* 1997; Diogon *et al.* 2007; Chan *et al.* 2015). Decreased lateral contraction in these strains could be compensated by a simultaneous increase in dorsoventral force. This leads to a hypothesis that, in the double mutants, the major contractile force shifts from in lateral to dorsoventral cells to result in near-normal elongation. Dorsoventral contraction could be triggered by either PAK-1 and/or Rho independent LET-502 activity (Truebestein *et al.* 2015).

Evidence of tissue-specific requirements for *let-502*, *mel-11*, and *pak-1* are currently indirect, based on phenotypes and expression patterns as outlined above. In this study, we test the model of discrete gene requirements in lateral *vs* dorsoventral epidermal cells by using tissue-specific rescue of *let-502* (expected to function laterally), and *mel-11* and *pak-1* (predicted to act dorsoventrally). We found sufficiency of lateral *let-502* but did not detect *mel-11* or *pak-1* dorsoventral rescue of their mutants. As a complementary assessment of tissue-specific gene function, we surveyed 10 single and double mutations to see if elongation can occur without the usual lateral cell contraction in cases where force is predicted to shift dorsoventrally. We found few cases of this. We conclude that the system is robust to mutation and many elongation genes likely function in both lateral and dorsoventral cells rather than being restricted to one or the other population.

## Materials and methods

### Strains and alleles

Wild-type *C. elegans* (N2, var. Bristol) were maintained on OP50 *Escherichia coli* on Nematode Growth Media (NGM) (Brenner 1974). Stocks were maintained at 15°, while temperature-sensitive (*ts*) mutant phenotypes were observed after upshifting L4 animals overnight to the nonpermissive temperatures. A list of mutant alleles and transgenic strains can be found in Supplementary Table S1. Information on genes, alleles, and promoters involved in this project can be found at Wormbase.org.

### Genomic DNA, RNA, and cDNA isolation

cDNA was used for tissue-specific expression of *let-502*, *pak-1*, and *mel-11* to ensure that intronic regulatory regions would not interfere with functional expression in lateral or dorsoventral tissue, and to reduce the size of expression constructs. *let-502* cDNA was isolated from a total wild-type RNA pool. *pak-1* and *mel-11* cDNA-containing plasmids were supplied by Dr. Y. KOHARA (National Institute of Genetics, Japan) and isolated using standard PCR. A list of primers used to isolate *let-502*, *pak-1*, and *mel-11* cDNA can be found in Supplementary Table S2. Primers were designed using Primer3 and Oligocalc interfaces.

Genomic DNA was isolated from wild-type worms using lysis with Protease K (He 2011a). Total RNA was isolated using a Trizol-based protocol (He 2011b) and genomic DNA contamination was reduced with DNAse I (New England BioLabs). *pak-1* and *mel-11* cDNAs were isolated using a High-Capacity cDNA Reverse Transcription kit with random hexamer primers (Thermo Fisher Scientific). *let-502* cDNA was generated with primers oED1, oED2, oED36, and oED37 (Supplementary Table S2). *let-502* could not be isolated as one product, so fragments were isolated and stitched together (oED1 and oED2) using PCR.

### Tissue-specific rescue constructs

Tissue-specific rescue constructs were used to determine sufficiency of expression in lateral (*pceh-16* promoter) (Cassata *et al.* 2005) or dorsoventral (*pelt-3* promoter) (Gilleard *et al.* 1999) epidermal tissue. The cDNA of *let-502* (3.5 kb, coding sequence C10H11.9.1), *pak-1* (1.7 kb, coding sequence, C09B8.7a.1), and *mel-11* (3 kb, coding sequence C06C3.1a.1) were cloned into pBluescript II KS(+) (2.9 kb). Primers used are listed in Supplementary Table S2 and restriction enzymes used in cloning are found in Supplementary Table S3. Endogenous promoters (*plet-502* [5 kb upstream of the first ATG], *ppak-1* [5.3 kb], and *pmel-11* [4 kb]) and cell-specific rescue promoters (*pceh-16* [2.9 kb] and *pelt-3* [1.9 kb]) were cloned upstream of the cDNAs. *gfp* (1 kb) and *unc-54* 3’ UTR (305 bp) were cloned downstream of the cDNAs. Constructs were confirmed with restriction enzyme digestion and by DNA sequencing (University of Calgary DNA Services) to confirm that the genes were in frame.

Plasmids were injected at 5 ng/µl. For *let-502* constructs, pRF4 [*rol-6*(*su1006*), 50 ng/µl] (Mello *et al.* 1991) and pJM67 (*elt-2::gfp*, 10 ng/µl) (Fukushige *et al.* 1999) served as transformation markers. For the *pak-1* constructs, 20 ng/µl with pCFJ90 (*myo-2::mCherry*, 2.5 ng/µl) or pTG96 (*sur-5::gfp*, 50 ng/µl) were used as transformation markers (Gu *et al.* 1998). For *mel-11*, 10 ng/µl with pRF4 [*rol-6*(*su1006*), 50 ng/µl] and pJM67 (*elt-2::gfp*, 10 ng/µl) or only pRF4 [*rol-6*(*su1006*), 50 ng/µl] served as transformation markers. pBluescript II KS(+) was added to each injection mixture to make a final DNA concentration of 100 ng/µl. Plasmid mixtures were injected into *let-502(ts)* (HR1157) for *let-502* constructs or *pak-1; let-502(ts)* (HR1500) for *pak-1* constructs and both were then maintained at the permissive temperature. The balanced strain *mel-11(ts) unc-4*/*mnC1* (HR604) was used for injecting *mel-11* constructs. Stable transgenic lines are listed in Supplementary Table S4. The GFP tags inserted 3’ to the cDNAs were not visible in the epidermis of our transformants, even for those that rescued. The previously documented CAN neuron expression for *pak-1* using its native promoter (Iino and Yamamoto 1998) was seen. The expected junction between the *pak-1* cDNA and the *gfp* inserted 3’ to it was confirmed by PCR to demonstrate incorporation of constructs into extragenic arrays in nonrescuing *pak-1* strains.

### Light, Nomarski, and fluorescence microscopy

Rescue of *let-502* and *pak-1* transgenic constructs was scored by measuring body length. Animals were upshifted to 24° or 25° as L4 larvae, and embryos were laid by adults the next day over a 2 hour period to produce semi-synchronized cohorts. Embryos were incubated for 24 or 48 hours before being photographed with a Canon G700 Camera mounted to a Zeiss Stemi SV 11 fluorescence dissecting microscope. Nomarski microscopy was used to image *pak-1; let-502* transgenic lines at higher magnification (40X) to score rescue to the twofold stage. Embryos were collected after 2 hours of laying and photographed 12 hours later. Larval lengths were measured with ImageJ, and data assessed with Mann-Whitney Rank Sum statistical tests from Prism software rather than *t*-tests due to nonnormality of distributions. *mel-11* rescue animals were scored via hatching rates.

Measurements of lateral cell width were made by collecting embryos from NGM plates or by cutting gravid adults in a drop of dH_2_O. Embryos were mounted on a Polylysine-coated slide and freeze-cracked followed by fixation in MeOH and Acetone, and staining with antibody to the membrane junctional marker MH27/AJM-1 antibody (Francis and Waterston 1991). Embryos were imaged at 40X using a Zeiss Axioplan 2i equipped with a Hamamatsu Orca ER camera in a single plane. Measurements of cell membrane and embryo lengths were made using ImageJ. Slopes of lines fitted by linear regression of lateral cell membrane length *vs* fold elongation were compared using two-tailed testing with Prism software.

### Data availability

Strains and plasmids are available upon request. The authors affirm that all data necessary for confirming the conclusions of this article are represented fully within the article and its Tables and Figures. Supplementary material is available at figshare: https://doi.org/10.25387/g3.14537895.

## Results

### LET-502/Rho kinase sufficiency in lateral *vs* dorsoventral epidermal cells

Actin microfilaments in lateral epidermal cells contribute the major contractile force for elongation, while the dorsoventral cells create tension against which this force pulls (Priess and Hirsh 1986; Diogon *et al.* 2007; Vuong-Brender *et al.* 2016). Asymmetric activity of *let-502*, *pak-1*, and *mel-11* may contribute to the asymmetric contractile properties of the two cell populations. To explore this idea, cell-specific promoters for lateral (*pceh-16*, Cassata *et al.* 2005) and dorsoventral (*ppelt-3*, Gilleard *et al.* 1999) epidermal cells were utilized to limit expression of *let-502*, *pak-1*, and *mel-11* to the desired cells. These promoters have been utilized extensively to demonstrate lateral function of *rhgf-2*, *mlc-4*, and *fhod-1*, dorsoventral sufficiency for *fem-2* and *rga-2* and to show that *hmp-1* can act in either tissue (Diogon *et al.* 2007; Gally *et al.* 2009; Chan *et al.* 2015; Refai *et al.* 2018; Vuong-Brender *et al.* 2018).

A caveat of this approach is that the multicopy transgenic arrays, we use often show only zygotic expression and are also subject to somatic mosaicism. Although *let-502* and *mel-11* are expressed both maternally and zygotically, zygotic expression is sufficient for normal elongation (Wissmann *et al.* 1997, 1999; Piekny *et al.* 2000). These types of experiments show where a gene is sufficient for rescue, but this could differ from where a gene acts under normal conditions.

Previous evidence suggests that *let-502*/Rho kinase is likely required laterally (see Section Introduction). Transgenic worms with either the lateral or dorsoventral promoters driving the *let-502* cDNA were generated in the temperature-sensitive *let-502(sb118)* background [hereafter referred to as *let-502(ts)*, Supplementary Table S4]. At 25°, *let-502(ts)* embryos hatch with early elongation phenotypes that show incomplete penetrance and expressivity (Vanneste *et al.* 2013). L4 hermaphrodites from the transgenic lines were plated overnight at 25°, allowed to lay embryos for 2 hours to produce a semi-synchronous brood, and embryos were incubated for another 24 hours after which larval body lengths were measured.

**Figure 2:**
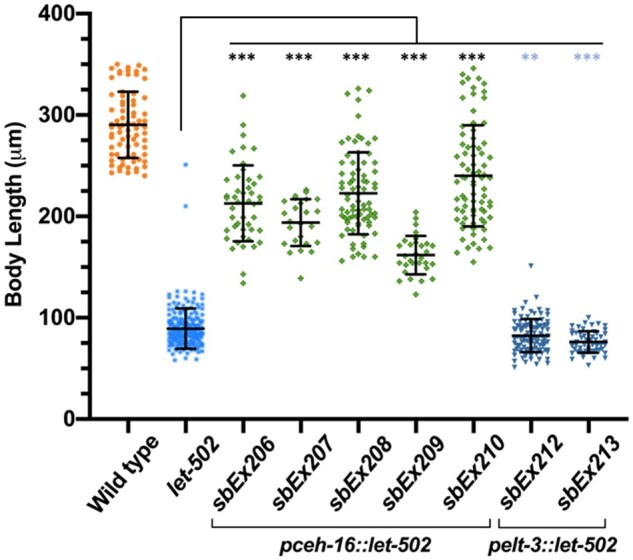
Rescue of body length when *let-502* was expressed in lateral but not dorsoventral epidermal cells. *let-502* cDNA expressing constructs were driven in lateral (*pceh-16*) or dorsoventral (*pelt-*3) epidermal cells in *let-502(ts)* mutant animals at 25°. Larvae were measured 24 hours after egg-laying. Expression of *let-502* in lateral epidermal cells rescued embryonic growth in all five lines. Expression in two dorsoventral epidermal cells were actually shorter than nontransgenic controls. Statistical significance relative to the nontransgenic *let-502(ts)* control was assessed with the Mann-Whitney Rank Sum test. Mean standard error bars are shown. ***, *P* < 0.0001; **, *P* = 0.002. Significance denoted by blue asterisks indicate that larvae were shorter, rather than longer, than control.

Transgenic worms using *let-502* cDNA driven by the lateral *pceh-16* promoter showed rescue in all five independent lines (*sbEx206*, *sbEx207*, *sbEx208*, *sbEx209*, and *sbEx210*) that were all significantly different from the *let-502(ts)* parent (*P* ≤ 0.0001 for all lines, Figure 2). All animals that did show rescue expressed the Roller phenotype that was used as a co-transformation marker. Conversely, transgenic worms expressing *let-502* under the control of dorsoventral *pelt-3* (*sbEx212* and *sbEx213*) showed no rescue. Although statistically different from the control, these lines were slightly shorter, rather than longer, than nontransgenic *let-502(ts)*. Thus, only *pceh-16* driven lateral *let-502* appeared sufficient for elongation.

### MEL-11/myosin phosphatase sufficiency in lateral *vs* dorsoventral epidermal cells

**Figure 3:**
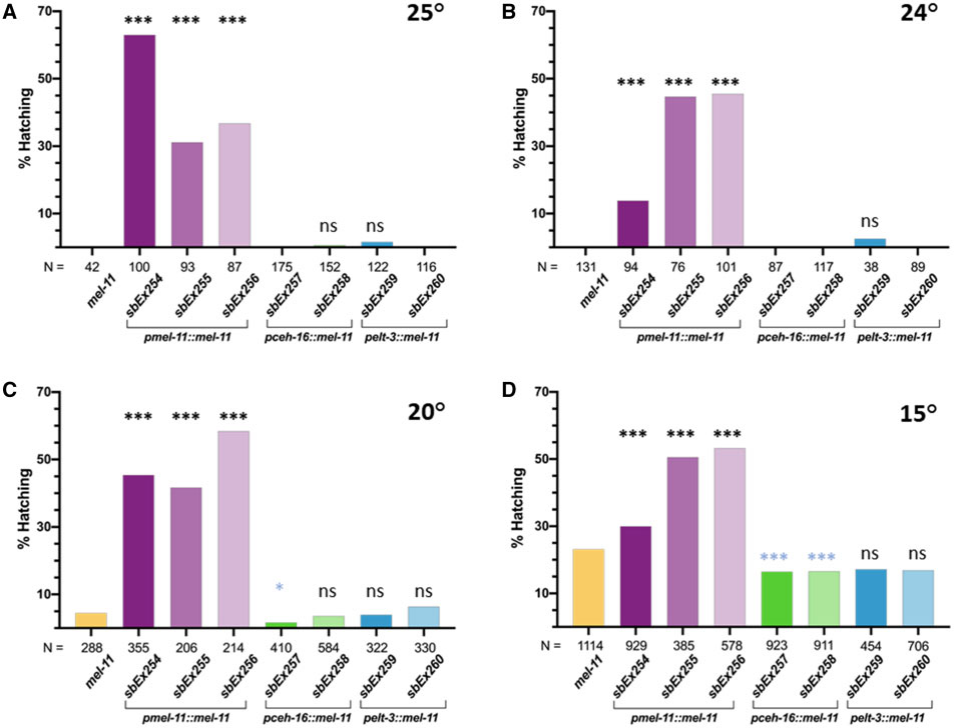
*mel-11* driven by its endogenous promoter rescues hatching while lateral or dorsoventral promoters do not. *mel-11* cDNA constructs were driven with the endogenous (*pmel-11*), lateral (*pceh-*16) or dorsoventral (*pelt-*3) promoters in *mel-11(ts)* animals. (A–D) Hatching was assessed at the indicated temperatures to compromise *mel-11(ts)* to different extents in an effort to detect weak rescue. While all three lines with the endogenous promoter rescued at all temperatures, hatching rates only increased sporadically above the control with lateral or dorsoventral transgenic lines. Significance *vs* the nontransgenic *mel-11* control was determined by chi-square with 1 degrees of freedom. ***, *P* < 0.0005; *,*P* = 0.03; ns, *P* > 0.05. Significance was not calculated in cases where hatch rates, like the control, were 0%. Significance denoted by blue asterisks indicate hatch rates that were lower, rather than higher, than the control. *N* values are shown for each experiment. While *N* = 42 for the *mel-11* control run in parallel at 25°, other experiments showed 0/1311 hatching embryos for this strain.

*mel-11* acts antagonistically with *let-502*, reducing actin contractility (Wissmann *et al.* 1997, 1999; Piekny *et al.* 2000) and we predict *mel-11* expression would be sufficient if expressed dorsoventrally. Extragenic transgenes were generated for *mel-11* cDNA driven by either the endogenous, *pceh-16* or *pelt-3* promoters and were examined for rescue of the *ts* allele *mel-11(it26)* [hereafter referred to as *mel-11(ts)*, Supplementary Table S4]. This mutation produces 100% unhatched embryos at 25° (Kemphues *et al.* 1988; Wissmann *et al.* 1997). Three independent *mel-11* cDNA lines driven by the endogenous *pmel-11* promoter (*sbEx254*, *sbEx255*, and *sbEx256*) rescued the 25° lethal phenotype with 63%, 31%, and 37% hatching, respectively (*P* < 0.0005, Figure 3A). This compared to 0/1311 hatched embryos for the nontransgenic control strain. Of the two *pceh-16* laterally driven transgenic lines, one showed 0.7% hatching (*sbEx258*) and the other showed none (*sbEx257*)*.* Similarly, of the two *pelt-3* dorsoventral driven transgenic lines, one showed 1.6% hatching (*sbEx259*) and the other (*sbEx260*) exhibited 0%.

Experiments were repeated at lower temperatures (24°, 20°, and 15°) in an effort to detect weaker effects, but once again, clear rescue was only observed for transgenic lines using the endogenous *pmel-11* promoter (Figure 3, B–D). Cases of statistical significance were for lower, rather than higher, hatching rates compared to controls. Our results may indicate that *mel-11* is only sufficient if expressed in both tissues or that the *pceh-16* and *pelt-3* promoters do not provide appropriate temporal or spatial control.

### PAK-1/p21-activated kinase sufficiency in lateral *vs* dorsoventral epidermal cells

**Figure 4:**
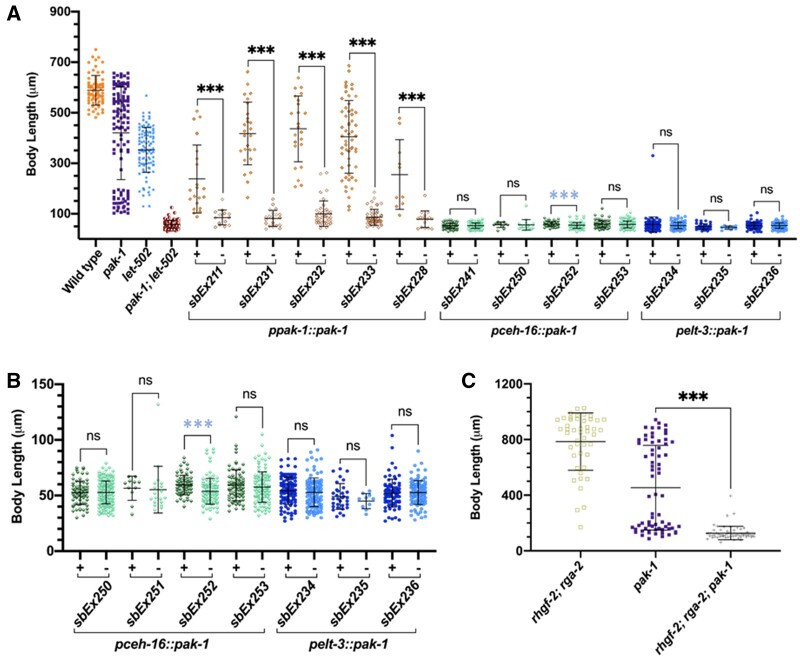
*pak-1* driven by its endogenous promoter rescued elongation but lateral and dorsoventral promoters did not. *pak-1* cDNA expressing constructs were driven with the endogenous (*ppak-1*) or tissue-specific promoters (*pceh-16*, lateral or *pelt-3*, dorsoventral) in the sensitized *pak-1; let-502(ts)* mutant animals at 24˚C. Larvae were measured 48 hours after egg-laying. Transgenic animals were distinguished based on *myo-2::mCherry*, *myo-2::gfp* or *sur-5::gfp* co-injection markers (Supplementary Table S4) and compared to their nontransgenic sibs. (A) *ppak-1* rescued early arrest of *pak-1; let-502(ts)* in five lines compared to their nontransgenic siblings. *pak-1* constructs driven in lateral (*pceh-16*, four lines) dorsoventral (*pelt-*3, three lines) did not rescue arrest. *sbEx252* was statistically significant, but transgenic animals were shorter. (B) As *pak-1* is required for late, muscle-driven elongation past the twofold stage, the same lines were observed at higher power (40x) to detect small differences in length to see if transgenes could rescue to the twofold stage. No rescue was observed with the lateral (*pceh-16*, four lines) or dorsoventral (*pelt-3*, three lines). *sbEx252* was statistically significant, but transgenic animals were shorter rather than longer. (C) *pak-1(+)* is required for the elongation that occurs in the *rhgf-2; rga-2* double mutant at 25°. Statistical significance was assessed with the Mann-Whitney Rank Sum test. Mean standard error bars are shown. ***, *P* < 0.0001; **, *P* = 0.002. Significance denoted by blue asterisks indicate that larvae were shorter, rather than longer than control.

In addition to the main contraction/elongation pathway involving *let-502* and *mel-11*, the *pak-1* pathway acts in parallel (Diogon *et al.* 2007; Martin *et al.* 2014, 2016). However, *pak-1(ok448)*, a likely null allele (Lucanic and Cheng 2008), has modest elongation defects (Gally *et al.* 2009; Martin *et al.* 2014), which would make detection of rescue difficult. *pak-1* is enhanced by *let-502(ts)* (Gally *et al.* 2009), and so we used a background sensitized with *let-502(ts)*. We previously used *let-502(ts)* to sensitize the genetic background of *fhod-1*, which like *pak-1* has impenetrant elongation defects, and we were able to demonstrate a lateral requirement for *fhod-1* (Refai *et al.* 2018). *pak-1* rescue was assessed at 24˚C, which is semi-permissive for *let-502(ts)* but results in a penetrant arrest phenotype in the double with *pak-1* (Figure 4A). Because *pak-1; let-502(ts)* larvae are slow growing, embryos were measured 48 hours after egg laying rather than the 24 hours used in Figure 2 for *let-502(ts)*. Transgenic animals were distinguished based on *myo-2::mCherry*, *myo-2::gfp* or *sur-5::gfp* co-injection markers (Supplementary Table S4) and compared to their nontransgenic sibs. Rescue was observed for *pak-1* cDNA driven by its endogenous promoter (*ppak-1*) across all five independent lines (*sbEx211*, *sbEx228*, *sbEx231*, *sbEx232*, and *sbEx233, P* < 0.0001, Figure 4A).

We did not observe tissue-specific rescue for *pak-1*. Three *pelt-3* dorsoventral lines (*sbEx234*, *sbEx235*, and *sbEx236*) and four *pceh-16* lateral lines (*sbEx241*, *sbEx250*, *sbEx252*, and *sbEx253*) showed little difference from nontransgenic sibs (Figure 4A). *sbEx252* showed statistical significance between transgenic and nontransgenic sibs, but transgenic animals were shorter rather than longer. It was possible that the transgenes rescued *pak-1*’s role during early elongation, but embryos then arrested at the twofold stage when the gene is again required, for muscle-mediated late elongation (Zhang et al. 2011). Unhatched embryos were observed at 40X using Nomarski microscopy 12 hours after egg laying. No rescue was observed (Figure 4B). The *sbEx252 pceh-16 *line was again statistically significant, but transgenic animals were shorter than their nontransgenic sibs. As we saw for *mel-11*, our results may indicate that *pak-1* is only sufficient if expressed in both tissues or that the *pceh-16* and *pelt-3* promoters do not provide appropriate temporal or spatial control.

### *rhgf-2, rga-2* requires *pak-1(+)*

We have previously shown that elongation in double mutants of *rhgf-2*/Rho GEF and *rga-2*/Rho GAP is dependent on *let-502*/Rho kinase (Chan *et al.* 2015), perhaps reflecting Rho independent LET-502/Rho kinase activity (Truebestein *et al.* 2015). *rhgf-2; rga-2* also requires the *pak-1* activator *fem-2* (Chan *et al.* 2015). To ask if this latter result reflects a need for *pak-1(+)* in *rhgf-2; rga-2*, we constructed *rhgf-2; rga-2; pak-1* triple mutants and measured their body lengths 2 days after egg laying at 25°. While few *rhgf-2; rga-2* showed retarded growth, about half of *pak-1* animals did so (Figure 4C). Virtually all the triple mutant *rhgf-2; rga-2; pak-1* arrested early, demonstrating that the near normal elongation in *rhgf-2; rga-2* is dependent on *pak-1(+)* as well as on *let-502(+)* and *fem-2(+)* as reported previously (Chan *et al.* 2015).

### Survey of elongation mutants reveals only minor changes in lateral cell shortening

To complement our tests for tissue-specific sufficiency, we examined mutants to gauge the necessity of gene function on lateral cell contraction. Our model of tissue-specific gene activity in Figure 1C leads to the following predictions. The lateral cells in mutants for the genes shown to be sufficient laterally (*rhgf-2, let-502*, and *fhod-1*) would contract proportionally less while mutants with decreased dorsoventral forces (*pak-1, pix-1*, and *fem-2*) would result in less rigid dorsoventral cells, allowing lateral cells to contract more (Figure 1C, columns 3 and 4, respectively, a more detailed discussion of the model is found in Supplementary Figure S1). Loss of dorsoventral inhibitors of contraction (*rga-2* and *mel-11*) would act as an impediment to lateral cell shortening (column 5). The strongest prediction is for double mutants of a lateral activator (*rhgf-2* or *let-502*) and a dorsoventral inhibitor (*rga-2* or *mel-11*). Here, we predict less lateral contraction as the force is transferred to the dorsoventral cells (column 6).

To survey a large number of strains, we used a method similar to Vuong-Brender *et al.* (2018). Epidermal cell membranes were marked by indirect immunofluorescence of fixed embryos stained with the MH27 antibody (Francis and Waterston 1991), which is directed against the junctional marker AJM-1. This approach has the advantage that many strains can be examined quickly and it obviates the need to introduce the AJM-1::GFP into each of the single and double mutant strains that we examined. Since large numbers of embryos can be scored rapidly for each strain, relatively rare subpopulations are potentially detected, which could be important for mutations with variable phenotypes. This approach is static, measuring lateral cell shortening *vs* embryo lengthening rather than lateral shortening *vs* time for live imaging.

**Figure 5:**
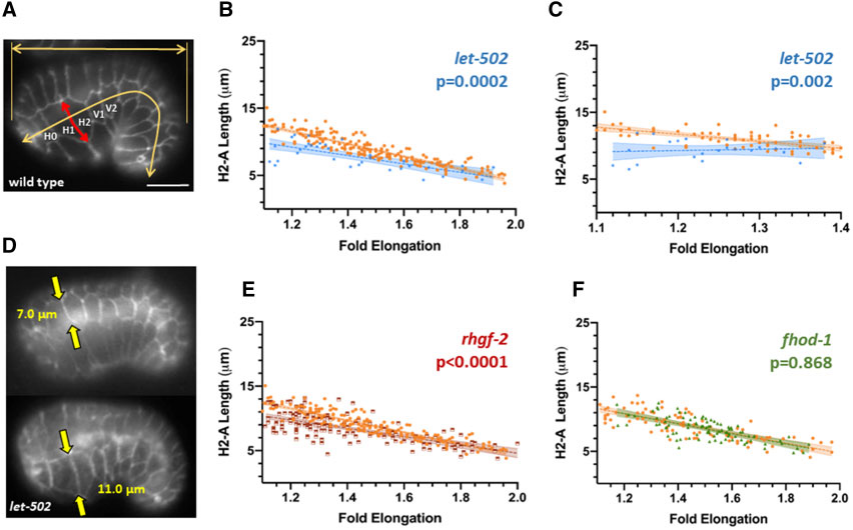
The H2-A membrane length as a function of fold elongation in embryos mutant for genes predicted to induce lateral contraction. (A) An embryo immunostained with the junction marker M27/AJM-1. The H2-A membrane is indicated by the red double headed arrow while the length of the embryo and long axis of the eggshell are indicated with yellow arrows. Fold elongation is the embryo length divided by that of the eggshell. Scale bar = 10 μm. (B, C, E, F) Orange symbols represent the wild-type control on each panel. Shading denotes 95% confidence intervals for the slopes of the regression lines and two-tailed *P*-values compare the slopes of mutant *vs* control. (B) *let-502(ts)* shows less shortening of the H2-A membrane at any given degree of elongation as indicated by different slopes. (C) *let-502(ts)* is shown up to the 1.4 stage, which excludes escapers of early arrest. A subset of embryos begin elongation with shorter H2-A membranes while others are in the wild-type range. (D) An example of two *let-502(ts)* embryos near the onset of elongation but with different H2-A lengths (yellow arrows). Note the lower embryo is slightly more advanced and yet has a longer H2-A membrane. Both embryos have unfused dorsal cells characteristic of early elongation, indicating that they did not retract in length from a later stage. (E) Mutants of the *let-502* activator *rhgf-2* showed a similar pattern to *let-502*, with a subpopulation of embryos with short H2-A membranes at the outset of elongation. (F) The lateral-specific actin nucleator *fhod-1* showed no apparent difference in slope from wild type.

We first determined which lateral cell boundary would be the most consistent metric for the analysis based on the correlation of shortening as a function of embryo elongation. Our focus was on the anterior of the embryo, which undergoes the greatest reduction of lateral cell width (Martin *et al.* 2016). The lengths of the membranes in the circumferential axis of cells H0, H1, H2, V1, and V2 were measured along with the length of the embryo curled up within the eggshell (Figure 5A). We also measured the length of the eggshell to account for differences in embryo size. We focused on the 1.1 (bean stage) to the twofold stage. The best linear correlation as a function of fold elongation was for the anterior membrane of H2 (*R*^2^ = 0.645, Supplementary Figure S2, A–E). We will refer to this membrane as H2-A (note that the anterior membrane of H2 is the posterior of H1). Wild-type embryos stained on different days were not statistically different (*P* = 0.113 for slope comparisons of regression lines, Supplementary Figure S2F). Martin *et al.* (2016) found that this membrane showed the most rapid rate of shortening, which was linear with time. Embryos with lateral cells facing directly upward or tilted to one side yielded similar correlations (Supplementary Figure S3, A and B) and so measurements were pooled.

#### Lateral contractile genes (*rhgf-2, let-502*, and *fhod-1*):

We predict that H2-A would contract relatively less in these mutants (Figure 1C, column 3). Martin *et al.* (2016) found that lateral cells shortened at a slower rate *vs* time for *let-502(RNAi)* as well as for the *sb118 let-502(ts)* allele that we use. As shown in Figure 5B, the slope of the regression line for *let-502(ts)* H2-A shortening *vs* fold elongation was shallower than wild type (*P* = 0.0002). This could imply that *let-502* lateral cell contraction contributes less to elongation than in wild type for any given length of the embryo. However, the population in Figure 5B includes leakers in the *ts* strain that elongate past the early arrest phase. Focusing on embryos of <1.4 fold enriches for more severely affected individuals (Figure 5C). In this range, it is apparent that the width of H2-A was variable, with some embryos at the beginning of elongation having H2-A widths equivalent to wild type at the twofold stage. Figure 5D shows an example of two *let-502(ts)* embryos at similar elongation stages with different H2-A lengths. This narrower H2-A width is the opposite of what is expected if *let-502* is necessary for the contraction of the lateral cells. Narrower than expected H2-A cells were also seen when the proportion of head width taken up by H2-A was plotted as a function of elongation (Supplementary Figure 3C). These phenotypes could reflect the gene’s earlier known function during ventral enclosure (Fotopoulos *et al.* 2013; Wernike *et al.* 2016; Wallace *et al.* 2018). It might appear that embryos with narrow H2-A cells may elongate without further lateral shortening, but the trajectory of individual embryos cannot be inferred from our static images.

RHGF-2 acts as the Rho GEF for LET-502/Rho kinase during early elongation and mutants of the two genes share similar phenotypes. Like *let-502* (Figure 3), lateral *rhgf-2* activity is sufficient for elongation (Chan *et al.* 2015). We used the hypomorphic allele *sb100* since homozygous null mutations show uniform early arrest. Like *let-502(ts)*, the *rhgf-2(sb100)* slope was shallower than wild type (*P* < 0.0001, Figure 5E), again with a subset of embryos having H2-A widths similar to wild type at the twofold stage.

*fhod-1* encodes a member of the formin class of actin nucleators and lateral expression is sufficient for elongation (Refai *et al.* 2018). However, *fhod-1* showed no change in slope compared to wild type in our assay (*P* = 0.868, Figure 5F). This is perhaps not surprising as only 15% of embryos with the null allele *tm2363* that we used show elongation phenotypes (Vanneste *et al.* 2013).

#### Dorsoventral contractile genes (*pak-1, pix-2, fem-2*):

**Figure 6:**
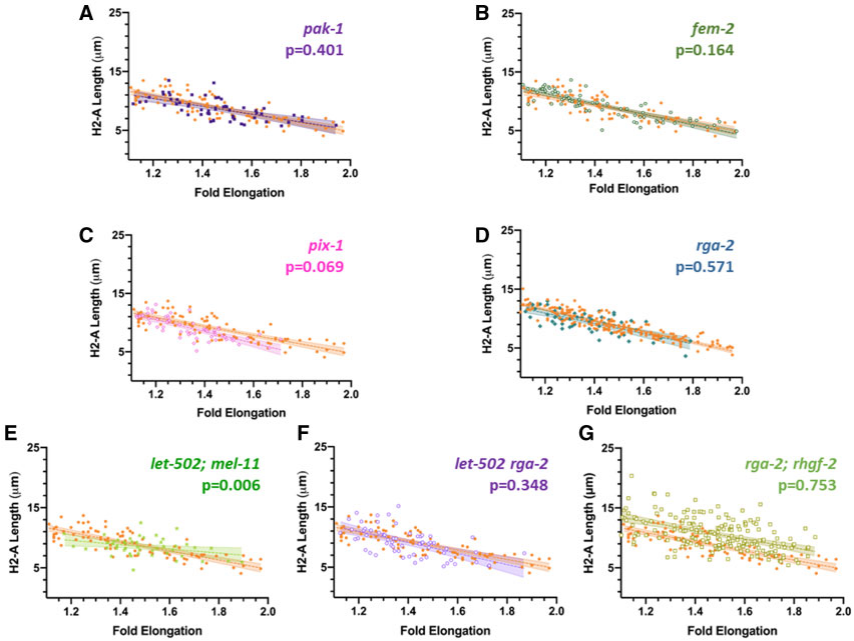
H2-A membrane length as a function of fold elongation in embryos mutant for genes predicted to induce dorsoventral contraction, inhibit lateral contraction, and multiply-mutant strains. The length of the H2-A membrane relative to fold length was determined as described in Figure 5A. In all panels, orange dots represent the wild-type control. Shading indicates 95% confidence limits for the slopes of the regression lines and two-tailed *P*-values compare the slopes of wild type *vs* mutant. Mutations in genes predicted to mediated dorsoventral contraction, *pak-1* (A), *fem-2* (B), and *pix-1* (C) did not alter the slope. A mutation in *rga-2* (D), which was expected to increase dorsoventral force, also resembled wild type. Double mutants that were thought to transfer contraction for lateral to dorsoventral cells were predicted to result in a shallower slope. This was true for *let-502; mel-11* (E), but not for *let-502 rga-1* (F), or *rhgf-2; rga-2* (G). *rhgf-2; rga-2* included individuals with narrow H2-A membranes at the onset of elongation, similar to *rhgf-2* single mutants (Figure 5E).

Loss of *pak-1* or its activators *pix-1* and *fem-2* are predicted to lessen dorsoventral forces and so lateral cells may contract more (Figure 1C, column 4). Slopes for *pak-1* and *fem-2* did not differ from wild type (*P* = 0.401 and 0.164, respectively, Figure 6, A and B). The slope of *pix-1* was slightly steeper as predicted (Figure 6C), with *P* = 0.069. Null mutations of these genes show impenetrant elongation phenotypes, therefore, their effects may be subtle.

#### Dorsoventral inhibitor gene (*rga-2*):

*rga-2* mutations result in dorsoventral hypercontraction and are predicted to have less H2-A shortening. However, we observed no slope difference compared to wild type (*P* = 0.571, Figure 6D).

#### Double mutants of lateral contractile genes (*let-502* and *rhgf-2*) and dorsoventral inhibitor genes (*mel-11* and *rga-2*):

The viability of these double mutant combinations, where lateral contraction may be lost while dorsoventral contraction is gained, might result in the contractile force shifting from lateral to dorsoventral. This leads to the prediction that in double mutants lateral cells would undergo relatively less contraction during elongation, resulting in a shallower slopes of their regression lines (Figure 1C, column 6). This was observed in *let-502; mel-11* (Figure 6E, *P* = 0.006) but not in *let-502 rga-2* (*P* = 0.348, Figure 6F). *rhgf-2; rga-2* also showed no differences (*P* = 0.753, Figure 6G). As for the *rhgf-2* single mutant (Figure 5E), many early embryos had narrower H2-A cells compared to wild type.

Overall, the genes expected to change the balance of forces between lateral and dorsoventral cells had at best weak effects, indicating that the embryo can adapt to those deficits.

## Discussion

Embryonic elongation of the *C. elegans* embryo up to the twofold stage is driven by changes in epidermal cell shape. This depends on multiple genes and interactions between those genes (Chisholm and Hardin 2005; Vuong-Brender *et al.* 2016; Carvalho and Broday 2020). Lateral and dorsoventral cells have distinctly different behaviors, with the former providing the contractile force and the latter producing tension against which this force pulls, to distribute force around the embryo (Priess and Hirsh 1986; Martin *et al.* 2016; Vuong-Brender *et al.* 2017). These differences are reflected by differential deployment of the actin cytoskeleton and at least parts of the Rho and Rac pathways in the two cell populations (Gally *et al.* 2009; Chan *et al.* 2015; Martin *et al.* 2016; Refai *et al.* 2018). Previously documented lateral or dorsoventral rescue (*rhgf-2, fhod-1, mlc-4, rga-2*, and *fem-2*) (Gally *et al.* 2009; Refai *et al.* 2018) or phenotypes (*let-502, pak-1, pix-1*, and *mel-11*) (Diogon *et al.* 2007; Martin *et al.* 2014, 2016) led to our hypothesis that there are generally discrete requirements for gene activity in either one or the other cell population (Figure 1, Supplementary Figure S1). This would explain why double mutants of genes thought to decrease lateral contraction with those that may function dorsoventrally to inhibit contraction suppress one another—the contractile force would switch from lateral to dorsoventral. The clearest example was for viability of double mutants of lethal mutations of the *let-502* activator, *rhgf-2*/Rho GEF and the *let-502* inhibitor *rga-2*/Rho GAP. The *rhgf-2* gene has been shown to be sufficient when expressed laterally while *rga-2* is sufficient when supplied dorsoventrally (Diogon *et al.* 2007; Chan *et al.* 2015). The dorsoventral force in *rhgf-2; rga-2* would then be supplied by either Rho independent LET-502 activity (Truebestein *et al.* 2015) and/or PAK-1 as wild-type alleles of both genes are required for *rhgf-2; rga-2* viability (Figure 4) (Chan *et al.* 2015). In this study, we test the model that elongation genes function either laterally or dorsoventrally but found that the system is more complex.

### Lateral *vs* dorsoventral rescue of *let-502*, *mel-11* and *pak-1*

We tested the prediction that *let-502* would be sufficient when expressed in the lateral epidermis while *mel-11* and *pak-1* would suffice when supplied dorsoventrally (Figure 1). To provide tissue-specific gene expression, we used the *ceh-16* and *elt-3* promoters, which have been previously used in *C. elegans* to demonstrate lateral *vs* dorsoventral requirements for *rhgf-2*, *mlc-4*, *fhod-1*, *rga-2*, and *hmp-1* (Diogon *et al.* 2007; Gally *et al.* 2009; Chan *et al.* 2015; Refai *et al.* 2018; Vuong-Brender *et al.* 2018). While *let-502* cDNA expression was sufficient in lateral epidermal cells in (Figure 2), neither *pak-1* nor *mel-11* cDNA expression were sufficient either dorsoventrally or laterally (Figures 3 and 4). The nonrescuing *pak-1* and *mel-11* constructs were confirmed by sequencing and restriction digests prior to injection and the presence of *gfp* inserted into the 3’ end of the transgenes was confirmed in the transformed strains.

Failure to rescue *pak-1* or *mel-11* may reflect the limitations of the experimental system. Previously tissue-specific sufficiency for *rga-2, rhgf-2, mlc-4, fhod-1*, and *fem-2* did not result in complete rescue of the corresponding mutants, either quantitatively (not all animals were rescued), or qualitatively (although viable, rescued mutant strains were not always morphologically wild-type) (Diogon *et al.* 2007; Gally *et al.* 2009; Chan *et al.* 2015; Refai *et al.* 2018). Incomplete rescue could be due to mosaic expression of extragenic arrays, lack of maternal expression and/or the levels and timings of the tissue-specific arrays being suboptimal. While *mel-11* and *pak-1* mutants were rescued by their native promoters, we were unable to generate the corresponding *pceh-16* and *pelt-3* double transgenic controls for either gene, either by multiple attempts of co-injection or crossing strains with single arrays to each other (data not shown). This failure may indicate toxicity when the genes are not under the fine control of the native promoters. For example, Martin *et al.* (2014, 2016) found that *pak-1* and *pix-1* phenotypes did not affect all dorsoventral cells similarly, with phenotypes being primarily anterior and dorsal. In contrast, the *pceh-16* and *pelt-3* promoters show uniform expression along the body axis (Gilleard *et al.* 1999; Cassata *et al.* 2005).

While negative outcomes must be treated with caution, failure to rescue *mel-11* and *pak-1* could indicate that the genes are required in both dorsoventral and lateral cells. Although *mel-11* phenotypes are prominent in dorsoventral cells (Diogon *et al.* 2007), the gene is also expressed, albeit at lower levels, in lateral cells (Wissmann *et al.* 1999; Piekny *et al.* 2003). The higher dorsoventral expression of *mel-11* could lead to stronger phenotypes in those cells, obscuring necessary functions in the lateral cells. Similar conclusions were drawn with the loss of function approach described below.

### Survey of the effects of elongation genes on lateral cell shortening

As an independent test of lateral *vs* dorsoventral specific gene requirements, we examined mutant strains predicted to transfer contractile force from lateral to dorsoventral cells. This would result in less shortening of the lateral cells for any given amount of embryo lengthening as the dorsoventral cells contracted more (Figure 1). This assesses the necessity, rather than the sufficiency of a gene’s function as seen using tissue-specific promoters. The experiments were carried out on embryos fixed and stained with the cell junction marker MH27/AJM-1 (Francis and Waterston 1991). Rather than measuring the rates of lateral cell contraction and lengthening of the embryo over time as in live imaging [which is slower in mutants like *let-502* (Norman and Moerman 2002; Diogon *et al.* 2007; Martin *et al.* 2014)], we measured how much the embryo lengthens for a given amount of lateral contraction. Ten different single and double mutant backgrounds could be quickly examined without having to introduce AJM-1::GFP into each strain. We focused on the anterior membrane of the H2 lateral cell (H2-A), which showed the most consistent correlation of shortening with elongation in wild type (Supplementary Figure S1). This membrane also undergoes the greatest amount of shortening (Martin *et al.* 2016; Vuong-Brender *et al.* 2018).

We found that mutants of *let-502*/Rho kinase and its activator *rhgf-2*/Rho GEF, which were predicted to decrease lateral contraction, did result in relatively less contraction for a given amount of embryo shortening (Figure 5). However, these mutations also produced a subset of embryos that began elongation with already shorter lateral cells. This phenotype likely reflects the known function of the *let-502* pathway during the earlier phase of ventral enclosure, the epiboly-like migration of epidermal cells from the dorsal surface to the ventral midline that encases the embryo in a continuous epidermal sheet (Fotopoulos *et al.* 2013; Wernike *et al.* 2016; Wallace *et al.* 2018).

Other genes that we surveyed did not give the predicted results, including *fhod-1, pak-1, pix-1*, *fem-2*, and *rga-2* (Figures 5 and 6). Our negative results could be attributed to impenetrant defects of some of the mutants. Double mutants that decreased lateral contraction while increasing dorsoventral force might be expected to have more dramatic effects by transferring contraction from one tissue to the other. We did find statistically significant effects for *let-502; mel-11*, but not for *let-502 rga-2*. The strongest prediction for our model was for double mutants of *rhgf-2* and *rga-2*, where lateral and dorsoventral sufficiency, respectively, had been previously demonstrated (Diogon *et al.* 2007; Chan *et al.* 2015). However, the predicted decreased reliance on lateral shortening was not observed.

Collectively, our results argue against the simple model that early elongation can be accounted for entirely by discrete functions confined to either lateral or dorsoventral cells. Elongation appears to be robust to mutation, likely indicating extensive redundancy beyond the known parallel actions of *let-502*/Rho kinase and *pak-1*/p21 activated kinase. While rescuing activity for some of the genes involved in early elongation has been demonstrated in one or the other cell population, sufficiency does not always reflect necessity of gene function. Indeed, even though *let-502, fhod-1, rhgf-2*, and *rga-2* have been shown to be sufficient either laterally or dorsoventrally, the corresponding proteins are expressed in both cell populations where their gene products may have a role (Wissmann *et al.* 1999; Piekny *et al.* 2003; Diogon *et al.* 2007; Vanneste *et al.* 2013). For example, as a cell changes shape, genes may function transiently to facilitate the redeployment of the contractile apparatus from areas where the cell is shrinking to areas where it is expanding. While any given gene may have its greatest effects either dorsoventrally or laterally, it is likely that most genes have critical roles in both groups of cells.
